# 甲磺酸伏美替尼一线治疗1例*EGFR* 20号外显子插入突变肺腺癌患者

**DOI:** 10.3779/j.issn.1009-3419.2024.102.11

**Published:** 2024-03-20

**Authors:** Zhengguo LI, Ting WEI, Duo ZENG, Li ZHAO, Jianting ZHANG, Laixiu CHEN

**Affiliations:** 733000 武威，甘肃省武威肿瘤医院呼吸内科; Department of Respiratory Medicine, Gansu Wuwei Cancer Hospital, Wuwei 733000, China

**Keywords:** 肺肿瘤, 甲磺酸伏美替尼, 表皮生长因子受体, 20号外显子插入突变, Lung neoplasms, Furmonertinib mesylate, Epidermal growth factor receptor, Exon 20 insertion mutation

## Abstract

随着基因组学、蛋白组学和分子生物检测技术的不断创新，晚期非小细胞肺癌（non-small cell lung cancer, NSCLC）的治疗模式逐渐多样化，已经从传统的化疗逐渐过渡到免疫治疗及靶向治疗。其中，以靶向酪氨酸激酶通路为靶点的分子肿瘤标志物在临床中发挥着越来越重要的作用。针对NSCLC患者表皮生长因子受体（epidermal growth factor receptor, EGFR）突变阳性，现阶段市面上已经有众多一线治疗药物上市，且均有较为明显的治疗效果。在中国患者中EGFR常见的变异位点位于19、20和21号外显子上，其中19和21号外显子突变是较为常见的突变类型，除此以外，EGFR突变还有一种亚型，被称为EGFR 20号外显子插入（EGFR exon 20 insertion, EGFR 20ins）突变。我们针对1例EGFR 20ins突变肺腺癌患者接受甲磺酸伏美替尼治疗进行总结，以期为临床诊疗提供有效借鉴。

## Abstract (en)

With the continuous innovation of genomics, proteomics and molecular biological detection technology, the treatment of non-small cell lung cancer (NSCLC) has changed from traditional chemotherapy to immunotherapy and targeted therapy. Among them, molecular tumor markers targeting tyrosine kinase pathways play more important roles in clinical practice. For advanced NSCLC patients with positive epidermal growth factor receptor (EGFR) mutations, there are many first-line drugs on the market and they could bring significant efficacy, thus completely subverting the treatment pattern of advanced NSCLC. Common mutations of EGFR in Chinese patients are located on exons 19, 20 and 21, of which exons 19 and 21 mutations are the more common types. Besides, there is also a subtype of EGFR mutations, known as EGFR 20 exon insertion (EGFR 20ins) mutation. The authors summarized the treatment of a lung adenocarcinoma patient with EGFR 20ins mutation accepting Furmonertinib mesylate, in order to provide effective references for clinical diagnosis and treatment.

研究数据^[1]^显示，肺癌是男性发病率和死亡率均为第一的恶性肿瘤，是女性发病率第二（仅次于乳腺癌）、死亡率第一的恶性肿瘤。中国肺腺癌患者中，表皮生长因子受体（epidermal growth factor receptor, EGFR）突变率可高达50.2%，19号外显子缺失突变最为常见，其次是21号外显子L858R点突变，分别占24.3%和22.9%^[2]^。近年来随着基因检测技术的持续进步和新兴药物的研发推进，针对EGFR敏感突变的非小细胞肺癌（non-small cell lung cancer, NSCLC）患者，奥希替尼等EGFR-酪氨酸激酶抑制剂（EGFR-tyrosine kinase inhibitors, EGFR-TKIs）的应用显著延长了此类患者的总生存期（overall survival, OS），已成为EGFR敏感突变NSCLC患者的标准治疗^[3⇓-5]^。但EGFR-TKIs对EGFR敏感突变之外的EGFR罕见突变的疗效差异较大。EGFR 20号外显子插入（EGFR exon 20 insertion, EGFR 20ins）突变是中国肺腺癌患者EGFR突变的第三大变异类型，占2.4%^[6]^，患者对奥希替尼等EGFR-TKIs的总体治疗反应不佳，尚存在未满足的临床需求。本文对1例甲磺酸伏美替尼在EGFR 20ins突变肺腺癌中的应用效果及安全性进行了总结，以期对临床诊疗有所帮助。

## 1 病例资料

患者，男性，54岁，因“肺癌术后复查”于2022年2月18日就诊于我院。患者2022年1月19日因刺激性咳嗽于衡阳市中心医院就诊，行胸部增强计算机断层扫描（computed tomography, CT）：右肺下叶后基底段结节，性质待定。肿瘤标志物示：癌胚抗原（carcinoembryonic antigen, CEA）升高（8.44 ng/mL），糖类抗原125（carbohydrate antigen 125, CA-125）、神经元特异性烯醇化酶（neuron-specific enolase, NSE）、细胞角蛋白19片段（cytokeratin 19 fragment, CYFRA21-1）、鳞状细胞癌相关抗原（squamous cell carcinoma antigen, SCC）均正常。查无明显手术禁忌证后，于2022年1月24日在衡阳市中心医院行胸腔镜下右肺切除+右中叶楔形切除+胸膜切除+纵隔胸膜切除+胸腔灌注化疗术（具体化疗药物及剂量不详），术后病检（病理号：3760520）示：（右下肺肿块）肺腺癌，肿瘤大小2.5 cm×2.0 cm×1.0 cm，被膜见癌侵犯，淋巴结转移（16/46）；术后诊断：腺癌，右肺恶性肿瘤，纵隔淋巴结继发恶性肿瘤，胸膜继发恶性肿瘤，pT2N2M1a IVA期，卡氏体能状态（Karnofsky performance status, KPS）评分为80分。

患者于2022年2月18日术后25 d在我院复查胸部增强CT，可见胸膜结节和胸膜下结节及右侧胸腔积液，于2022年2月22日在我院通过气管镜取得活检组织，并通过聚合酶链式反应（polymerase chain reaction, PCR）行基因检测，2022年3月2日结果显示为EGFR 20ins（DX1902）突变阳性。2022年3月4日完善正电子发射计算机断层显像（positron emission tomography/CT, PET/CT）检查，与胸部增强CT所见一致。经院内多学科会诊（multi-disciplinary treatment, MDT）后，2022年3月7日开始给予患者口服伏美替尼片80 mg qd。2022年3月22日为患者服药后首次复查，患者未诉不适症状，未出现治疗相关不良反应（treatment-related adverse events, TRAEs），影像学检查较前大致相仿，根据实体瘤疗效评价标准（Response Evaluation Criteria in Solid Tumors, RECIST）疗效评价为疾病稳定（stable disease, SD）。此后患者持续规律服用伏美替尼治疗，服药期间未诉不适症状，未报告TRAEs。2023年2月10日，患者再次就诊于我院，复查PET/CT，与上一次PET/CT（2022年3月4日）比较显示：右侧纵隔胸膜结节，代谢增高，较前体积略增大，右侧胸膜增厚，右侧胸腔积液（图1）。进一步完善胸腔彩超示：右侧胸腔存在一定量的积液（少量，超声见最大液深29 mm）。定位后行胸腔穿刺术，共抽出血性液体约450 mL，胸水CEA示：79.4 ng/mL，胸水脱落细胞学检查示：涂片中查见可疑癌细胞。因患者右侧纵隔胸膜结节较前体积略增大，而胸腔积液在治疗过程中持续存在，考虑肿瘤缓慢进展。动员患者重新抽取静脉血进行基因检测，未检测到基因突变。考虑靶向药物无法再次获益，与患者充分沟通后续治疗方案且结合患者意愿，患者拒绝继续服用靶向药物，患者于2023年2月26日停止口服伏美替尼，无进展生存期（progression-free survival, PFS）为11.0个月。后续患者于2023年3月23日开始行全身静脉化疗，方案为：卡铂注射液500 mg[曲线下面积（area under the curve, AUC）=5]，每周期第1天；培美曲塞二钠850 mg（500 mg/m^2^），每周期第1天，每21天为1个周期。截至2023年7月25日，患者已化疗6个周期，过程顺利，化疗期间未出现严重的TRAEs，KPS评分为90分，RECIST疗效评价为SD，疾病控制较好且药物应用安全性较好。

**图1:**
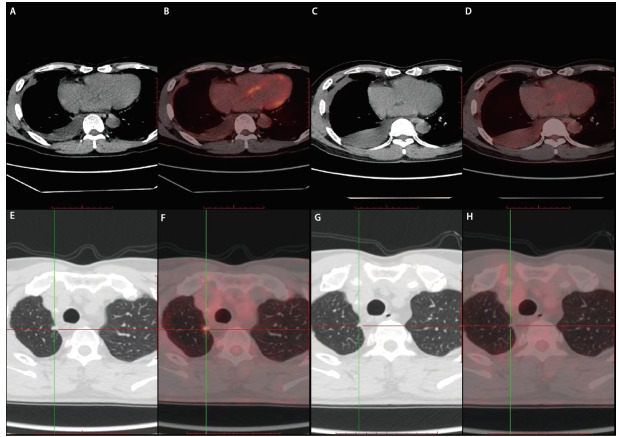
PET/CT结果比较。2023年2月10日PET/CT（A：右侧胸腔积液，软组织窗；B：右侧胸腔积液，代谢显像）与2022年3月4日PET/CT（C：右侧胸腔积液，软组织窗；D：右侧胸腔积液，代谢显像）对比，胸水量基本同前，但FDG药物摄取增高。2023年2月10日（E：纵隔胸膜结节，软组织窗；F：纵隔胸膜结节，代谢显像）与2022年3月4日（G：纵隔胸膜结节，软组织窗；H：纵隔胸膜结节，代谢显像）对比，纵隔胸膜结节较前略增大，代谢增高。

## 2 讨论

EGFR突变是NSCLC中最为普遍存在的致癌驱动突变之一，大约15%的白种人和近50%的亚洲晚期NSCLC患者存在EGFR突变^[7]^。EGFR基因突变主要见于18-21号外显子，其中EGFR 19号外显子del突变、21号外显子L858R点突变最为常见；而其他EGFR突变类型的突变频率低，一般定义为EGFR罕见突变或非经典突变。EGFR罕见突变中，最常见的类型是EGFR 20ins突变，常见于不吸烟者、女性等患者人群中。中国晚期肺腺癌患者中，EGFR 20ins突变占比达2.4%（合并或不合并其他EGFR突变），是仅次于EGFR 19del和L858R两大突变的“第三大突变”^[2]^。EGFR 20ins突变因其空间构型特殊，异质性强，一直以来缺乏安全有效的靶向治疗手段。研究^[8]^表明，对于伴有EGFR 20ins突变的NSCLC患者，接受一线以铂类为基础的化疗方案患者的中位PFS（6.4个月，95%CI：5.7-7.1）优于接受第一、二、三代EGFR-TKIs（2.9个月，95%CI：1.5-4.3，P<0.001）及仅接受第一代EGFR-TKIs的患者（2.0个月，95%CI：0.2-3.8，P<0.001）。

甲磺酸伏美替尼是由我国自主研发的第三代EGFR-TKIs，对EGFR敏感突变（19del/L858R）及耐药性T790M突变均有确切疗效^[9,10]^，已获得国家药品监督管理局（National Medical Products Administration）批准用于相应晚期NSCLC患者治疗。高剂量伏美替尼对多种EGFR罕见突变具有良好的抑制活性，已有多项临床研究报道了高剂量伏美替尼在EGFR 20ins突变晚期NSCLC患者中的疗效和耐受性。Han等^[11]^的研究显示，伏美替尼240 mg qd用于EGFR 20ins突变晚期NSCLC一线治疗，确认客观缓解率（objective response rate, ORR）为78.6%，疾病控制率（disease control rate, DCR）为100%，中位缓解持续时间（duration of response, DoR）达到15.2个月，已有患者持续接受治疗并获得疾病缓解超过26个月，且高剂量伏美替尼临床应用耐受性良好，仅有4%的患者出现3级及以上TRAEs，常见的TRAEs为腹泻、贫血、口腔溃疡、肝酶升高和血肌酐升高，没有患者因TRAEs导致治疗中止。Zhang等^[12]^的一项个案报道中，1例EGFR 20ins突变的IVB期（cT2aN3M1c）肺腺癌患者，接受伏美替尼（160 mg qd）二线治疗，PFS为10.0个月，DoR为8.0个月，总生存期为22.0个月，治疗效果显著，并且在治疗期间未出现严重的TRAEs。Sa等^[13]^的一项真实世界研究探讨了不同剂量伏美替尼治疗EGFR 20ins突变晚期NSCLC患者的疗效差异，53例患者接受伏美替尼作为一线或后线治疗，总体ORR为37.7%，DCR为92.5%，240 mg剂量组ORR为42.9%，优于160 mg剂量组的39.5%和80 mg剂量组的25.0%，但没有统计学差异（P=0.816），结果可能受到不同治疗线数的混杂影响。

本文报道了真实世界中伏美替尼80 mg qd一线治疗1例EGFR 20ins突变晚期NSCLC患者的临床疗效及安全性。患者在治疗期间未出现不适症状，未报告TRAEs，考虑肿瘤缓慢进展后停药并转为含铂双药化疗。截至2023年7月25日，患者仍在继续随访中，肿瘤状况尚稳定。伏美替尼治疗的成功可能为EGFR 20ins突变晚期NSCLC患者提供一种新的选择。

伏美替尼一线治疗EGFR 20ins突变晚期NSCLC的大型国际多中心III期注册临床研究（NCT05607550）以及伏美替尼二线治疗含铂化疗后EGFR 20ins突变晚期NSCLC的国际多中心II期临床研究（NCT05466149）均正在进行中，期待研究结果的公布。
